# Growth retardation and congenital heart disease in a boy with a ring chromosome 6 of maternal origin

**DOI:** 10.1186/s13039-022-00586-1

**Published:** 2022-03-05

**Authors:** Yanling Dong, Jian Li, Ziye Zeng, Xue Zhang, Mingxin Liang, Hong Yi, Jianyun Luo, Junnan Li

**Affiliations:** Department of Obstetrics and Gynecology, The First Affiliated Hospital of Chongqing Medical University, No. 1, Youyi Road, Yuanjiagang, Yuzhong District, Chongqing, 400016 People’s Republic of China

**Keywords:** Ring chromosome 6 (RC6), SNP array, Prenatal diagnosis

## Abstract

**Background:**

Rare chromosomal structural abnormalities, including ring chromosomes, often pose challenges to clinical genetic counselling.

**Results:**

Here, we report a newborn with congenital heart disease and developmental delay who inherited ring chromosome 6 [46,XY,r(6)(p25q27)mat] from a phenotypically normal mother. Genotypes and phenotypes were analysed by molecular cytogenetic analysis, whole-exome sequencing and literature review.

**Conclusions:**

Our study showed that the pathogenicity of the ring chromosome abnormality [r(6)(p25q27)] was mainly affected by chromosome imbalance, deletions of genes with haploinsufficiency, duplications of genes with triple sensitivity, parental inheritance of the imbalance and the imprinting status of the affected genes.

## Background

Ring chromosomes (RC) are a specific chromosomal abnormalities, being rare genetic events caused by terminal deletions and an intrachromosomal fusion [1]. RCs were first discovered in tumour cells in 1956 [2] and later in other autosomal and sex chromosomes in clinical cases [3–7]. To date, all 23 human chromosomes have been reported to be involved in RC-formation, with an overall incidence between 1/30,000 and 1/60,000 [8]. Two main types of RCs have been described: (1) 46,XN,r, where normal linear homologues are replaced by full-length rings or unbalanced rings [8]; and (2) 47,XN,+r, where the RC is supernumerary. In both cases, RC-carrying cell lines may coexist with normal cell lines in the mosaic state.

At the time of publication of this article, there have been few reports about RCs derived from chromosome 6 [9]; inheritance from a parent was not reported yet.

Here, genome-wide copy number and pedigree analysis were performed on a foetus-to-newborn case by banding cytogenetics and molecular genetics, and a hereditary RC6 abnormality was identified [r(6)(p25q27)]. Clinical consequences and implications for genetic counselling are discussed here.

## Case report

A 23-year-old pregnant woman, G1P0 (gravida 1, para 0), was admitted to foetal medical centre. The pregnant woman was 142 cm tall, within weight in the normal range for height, as were her parents and husband. The couple had normal intellectual development and no abnormal family history or mutagenic exposures.

**Fig. 1:**
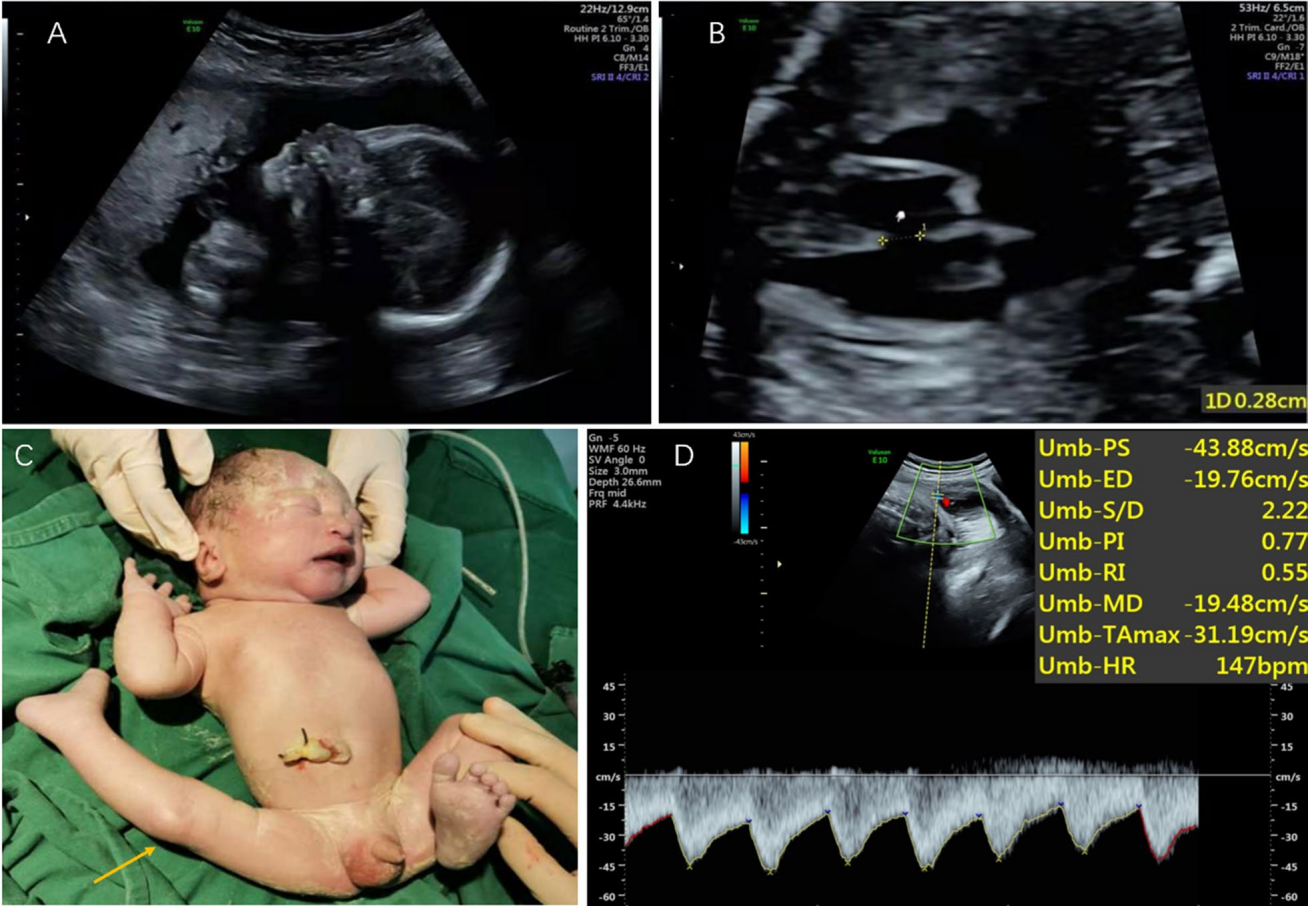
Foetal ultrasound at 24 weeks and abnormal newborn detections: absence of nasal bone (**A**) and ventricular septal defect (**B**). The right knee joint of the newborn was dislocated (**C**). Colour ultrasound indicated congenital heart malformation: ventricular septal defect; atrial septal defect (muscle) (**D**)

This pregnancy was conceived naturally. No noninvasive prenatal genetic testing (NIPT) was performed in the first trimester of pregnancy. However, sonography at 24+ weeks of gestation (w.o.g.) detected intrauterine growth retardation (IUGR), absence of nasal bone (Fig. 1A), and ventricular septum defect (Fig. 1B). Ultrasonography at 30^th^ w.o.g. confirmed the previous findings and additionally a *foramen ovale*. However, at 34 w.o.g. a second ultrasound examination revealed no abnormalities at all.

**Fig. 2:**
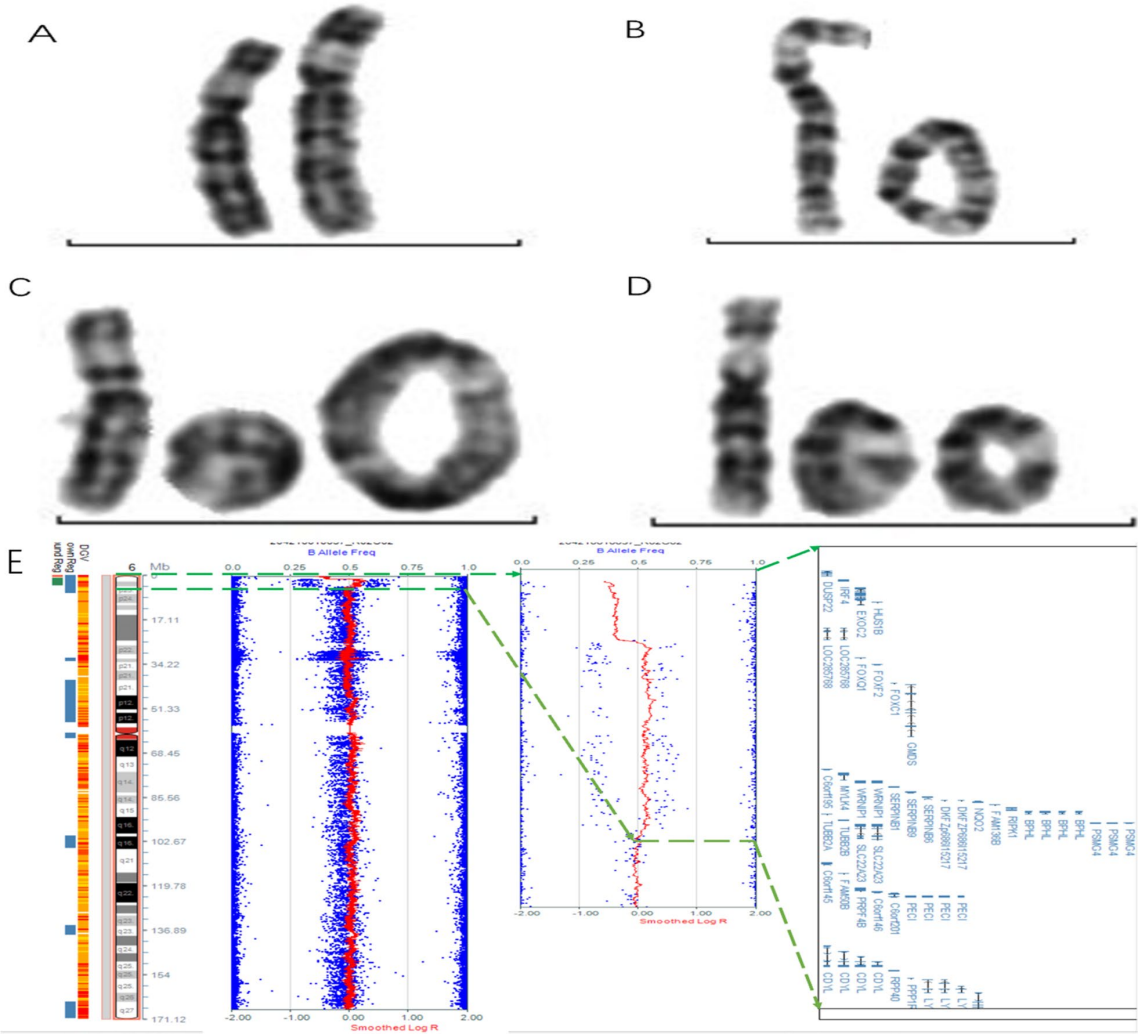
Examples of patient chromosome 6. **A** Normal chromosome 6. **B** Ring chromosome 6, r(6)(p25q27). **C** Double ring chromosome 6: r(6)(p25q27),+r(6)(p25q27). **D** Ring chromosome 6 and dicentric 6 ring chromosomes: r(6)(p25q27),+dic(6;6)(p25q27;p25q27). **E** SNP analysis of foetal uncultured amniocytes: arr[GRCH37] 6p25.3(203,254_1,138,134)×1,6p25.3p25.2(1,153,042_4,172,096)×3

Cytogenetic analysis (G-banding resolution was approximately 400–550 bands) and chromosomal microarray (CMA) were done after amniocentesis in 24+ w.o.g.. Also maternal blood sample and that of parents of the mother were cytogenetically analysed. After birth, karyotype and CMA analyses were performed again. Pre- and postnatal banding cytogenetics showed a karyotype of 46,XY,r(6)(p25q27)mat. The mother had in peripheral blood a mosaic karyotype: 46,XX,r(6)(p25q27)[44]/47,XX,r(6)(p25q27),+r(6)(p25q27)[2]/46,XX[15], and the father had a normal result as 46,XY (Fig. 2A–D). The karyotypes of the maternal grandmother and grandfather were normal (46,XX; 46,XY).

For CMA a SNP array was performed using KaryoStudio 1.4.3.0 Build 37 software (Illumina, San Diego, CA) to define possible copy number changes. Besides whole-exome sequencing (WES) was completed by the BGI Huada Gene Shenzhen Huada Clinical Testing Centre as previously reported [9]. Obtained molecular genetic data was bioinformatically analysed using DECIPHER (http://decipher.sanger.ac.uk), UCSC (http://genome.ucsc.edu), DGV (http://dgv.tcag.ca/dgv/app/home), ClinGen (http://dosage.clinicalgenome.org/), gene imprint database (http://www.geneimprint.com) and other Online-Mendelian Inheritance in Man (OMIM) databases (http://www.omim.org). Karyotype and CMA-results are described according to the International System for Human Cytogenomic Nomenclature (ISCN, 2020) [10].

CMA analyses in the foetus (amnion and peripheral blood) gave the following result: arr[GRCH37] 6p25.3(203,254_1,138,134)×1,6p25.3p25.2(1,153,042_4,172,096)×3 (Fig. 2E). In the mother the CMA-findings were: arr[GRCH37] 6p25.3(203,254_1,138,134)×1~2,6p25.3p25.2(1,153,042_4,172,096)×2~3. SNP-array confirmed the mosaic situation of 90% of the cells carrying the ring chromosome; also a isoUPD(6) mosaicism was found for 10% of the cells, explaining the 15 cells with normal karyotype 46,XX found in cytogenetics as being due to monosomic rescue.

Whole-exome sequencing confirmed the result of SNP-array as: seq[GRCh37] dup(6)(p25.3p25.2) chr6:g.1127408_4191151dup (3.06 Mb) and seq[GRCh37] del(6)(p25.3p25.3) chr6:g.63810_1127408del (1.06 Mb).

The boy was delivered by caesarean section at 39^+2^ weeks of gestation. Congenital dislocation of the right knee joint occurred in the newborn (Fig. 1C), even though no knee joint abnormality was observed at any stage of pregnancy. After treatment, the dislocation of the knee and limb was normal. The newborn had a birth weight too low for gestational age of 2.150 kg, and was overall in good mental condition, without any inborn defects. However, follow-up 8 months of age showed developmental delay concerning length (64 cm) and weight (5 kg); also congenital heart malformation was diagnosed by Doppler sonography as ventricular septal defect and atrial septal defect with the enlarged diameter of pulmonary artery and left heart enlargement; also the third top valve had a micro reflux and pulmonary hypertension was detected while left ventricular systolic function was normal (Fig. 1D).

Overall, as of the date of publication of this article, there have been no abnormal phenotypes in the newborn except for growth retardation and congenital heart malformations.

## Discussion

Here we report the first case of a maternally inherited RC6 r(6)(p25q27) without major clinical consequences. Yet, 9 cases have been reported in the literature with comparable de novo r(6)(p25q27), diagnosed between 2 and 13 years old. After 2013, molecular technology was applied to determine the breakpoint; for ring chromosome 6 with 6p25 to 6q27, all cases reported in the literature apart from the present one (Table 1) are de novo. Most patients have clinical features, including dysmorphic face, mental retardation, cerebellar malformation, delayed development, and cardiac abnormalities. The details of the genes involved in the chromosomal imbalance region [46,XY,r(6)(p25q27)] are shown in Table 2 and indicate that most of these genes are OMIM genes, such as *DUSP22, IRF4,* and *FOXC1*. There are currently two imprinted genes located on chromosome 6p25 (Table 2): *FAM50B* and *PXDC1*. Both genes were paternally expressed. Even though UPD(6) was detected in 10% of the blood cells of the mother of the patient, a clinical effect is not likely due to that postzygotic rescue phenomenon.

**Table 1:** Cases reported in the literature with r(6)(p25q27)

Year	PMID	Karyotype	Molecular technology	Parental karyotype	Duration of follow-up	Clinical phenotype
1990	2333874	46,XX,r(6)(p25q27)/46,XX	not apply	Normal	Born—13 years old	Facial abnormalities, mental retardation, epilepsy
1996	8905901	46,XX,r(6)(p25q27)/45,XY,-6/45,XY,-6,+f	not apply	The mother was normal and the father not provide it	Prenatal-17 months	Hydrocephalus, global retardation
2001	11223855	46,XY,r(6)(p25q27)/46,XY,dic r(6;6)(p25q27;p25q27)/45,XY,-6	not apply	The father was normal and in mother there was a Robertsonian translocation	Born—11 years old	Aortic root dilatation
2013	23398904	46,XY,r(6)(p25q27)	FISH + CMA	Not provided	sixteen months old	*Growth disorders, heart disease, facial abnormalities*
2015	26213576	46,XX,r(6)(p25q27)/46,XX,dic r(6;6)(p25q27;p25q27)/45,XX,-6	CMA	Not provided	*3 years old*	*Periventricular ectopia and white matter abnormalities*
2018	30305128	46,XY,r(6)(p25.3q27)/46,XY,dic r(6;6)(p25.3q27;p25.3q27)/45,XY,-6	FISH + CMA	Normal	*11 years old*	*Stunting, mental retardation, microcephaly*
2018	29656294	46,XY,r(6)(p25q27)/46,XY,dic r(6;6)(p25q27;p25q27)/45,XY,-6	FISH + CMA	*Normal*	*12 years old*	*Abnormal facial appearance, stunting, heterotopic gray matter*
2018	30225942	46,XY,r(6)(p25.3q27)	MLPA + CMA	*Not provided*	*Prenatal—2 years old*	*Anterior segment dysplasia and cardiac abnormalities*
2021	8504673	46,XX,r(6) (p25q27)	CMA	*Normal*	10 years old	Microcephaly, Abnormal facial appearance, hypertelorism, and cardiac abnormalities

*CMA* Chromosome Microarray Analysis; *FISH* Fluorescence In Situ Hybridization; *MLPA* Multiplex Ligation-dependent Probe Amplification

**Table 2:** Genes present in the 6p25.3 deleted region and 6p25.3p25.2 duplicated region

Gene	Description	Gene type	%HI	Imprinting status	Known syndromes/diseases	ID of OMIM
Genes present in the 6p25.3 deleted region						
*DUSP22*	dual specificity phosphatase 22	PC	38.68	NA	NA	616778
*IRF4*	interferon regulatory factor 4	PC	19.27	NA	Skin/hair/eye pigmentation, variation in, 8	601900
*EXOC2*	exocyst complex component 2	PC	34.26	NA	NA	615329
*HUS1B*	HUS1 checkpoint clamp component B	PC	97.37	NA	NA	609713
Genes present in the 6p25.3p25.2 duplicated region						
*BPHL*	biphenyl hydrolase like	PC	69.92	NA	NA	616778
*LINC01600*	long intergenic non-protein coding RNA 1600	ncRNA	99.38	NA	NA	NA
*C6orf201*	chromosome 6 open reading frame 201	PC	90.99	NA	NA	NA
*ECI2*	enoyl-CoA delta isomerase 2	PC	64.53	NA	NA	608024
*FAM217A*	family with sequence similarity 217 member A	PC	71.39	NA	NA	NA
*FAM50B*	family with sequence similarity 50 member B	PC	73.35	Imprinted (Paternal)	NA	614686
*FOXC1*	forkhead box C1	PC	9.01	NA	Anterior segment dysgenesis 3, multiple subtypes, AD; Axenfeld-Rieger syndrome, type 3, AD	601090
*FOXF2*	forkhead box F2	PC	29.64	NA	NA	603250
*FOXQ1*	forkhead box Q1	PC	74.58	NA	NA	612788
*GMDS*	GDP-mannose 4,6-dehydratase	PC	3.84	NA	NA	602884
*MYLK4*	myosin light chain kinase family member 4	PC	57.67	NA	NA	NA
*NQO2*	N-ribosyldihydronicotinamide: quinone reductase 2	PC	69.72	NA	Breast cancer susceptibility	160998
*PRPF4B*	pre-mRNA processing factor 4B	PC	3.38	NA	NA	602338
*PSMG4*	proteasome assembly chaperone 4	PC	70.84	NA	NA	617550
*PXDC1*	PX domain containing 1	PC	64.96	Imprinted (Paternal)	NA	NA
*RIPK1*	receptor interacting serine/threonine kinase 1	PC	52.24	NA	Autoinflammation with episodic fever and lymphadenopathy, AD;	603453
*SERPINB1*	serpin family B member 1	PC	35.07	NA	Immunodeficiency 57 with autoinflammation, AR	130135
*SERPINB6*	serpin family B member 6	PC	69	NA	NA	173321
*SERPINB9*	serpin family B member 9	PC	88.04	NA	?Deafness, autosomal recessive 91,AR	601799
*SLC22A23*	solute carrier family 22-member 23	PC	50.77	NA	NA	611697
*TUBB2A*	tubulin beta 2A class IIa	PC	20.26	NA	NA	615101
*TUBB2B*	tubulin beta 2B class IIb	PC	24.97	NA	Cortical dysplasia, complex, with other brain malformations 5, AD	612850
*WRNIP1*	WRN helicase interacting protein 1	PC	36.94	NA	Cortical dysplasia, complex, with other brain malformations 7, AD	608196

*AD* autosomal dominant; *AR* autosomal recessive; %*HI* DECIPHER Haploinsufficiency index (High ranks (e.g. 0–10%) indicate a gene is more likely to exhibit haploinsufficiency, low ranks (e.g. 90–100%) indicate a gene is more likely to NOT exhibit haploinsufficiency). *PC* protein-coding gene. *ncRNA* non-coding RNA. *NA* not accessible. *OMIM* (https://omim.org/): Online Mendelian Inheritance in Man®. ClinGen Haploinsufficiency Score: score of haploinsufficient (deletion) or triplosensitive (duplication) (https://dosage.clinicalgenome.org/)

RC formation mechanisms may include the loss and/or acquisition of genetic material. Previous studies have shown that at least three mechanisms may lead to RCs: inv dup del rearrangements, double-strand breaks and telomeric junctions [11]. RCs are generally considered to be the result of chromosomal aberrations during meiosis or in early postzygotic phase. Two open ends are connected to form a continuous ring. This mechanism assumes that some genetic material may be lost during ring formation. Also RCs tend to be lost during mitoses and cells with 45,XN,-6 are not viable. This is the reason for IUGR observed in the patient and his mother.

In conclusion, we reported the first case of a foetus with r(6)(p25q27).arr[GRCH37] 6p25.3(203,254_1,138,134)×1,6p25.3p25.2(1,153,042_4,172,096)×3 originating from the mother. Although other genetic effects on the congenital abnormity of the foetus cannot be excluded, the pathogenicity is mainly due to loss of RC6 during mitoses, leading to growth restrictions. Also influence of terminal deletion and duplication in chromosome 6 on heart phenotype cannot be excluded.

## Data Availability

The datasets used and/or analysed during the current study are available from the corresponding author on request.

## Abbreviations

CMA: Chromosomal microarray analysis
FGR: Foetal growth restriction
HGMD: Human Gene Mutation Database
RCs: Ring chromosomes
SNPs: Single-nucleotide polymorphisms
SNVs: Allele-nucleotide variants
WES: Whole-exome sequencing
